# Prognosis in Scarlet Fever

**Published:** 1896-12-05

**Authors:** J. O. Symes


### PROGNOSIS IN SCARLET FEVER.

J. O. Symes, M.D.

In view of the many complications which may arise, it is always necessary to exercise great caution in making a prognosis in this disease. There are, however, many prominent characteristics both of a general and clinical nature which are undoubtedly of use in helping us to form some idea of the future course of the malady. One of the most important of these is the question of age. Whitelegge found that the greatest number of children are attacked between the ages of four and five years, but that the mortality is highest during the second and third years of life. The curve of age mortality rises rapidly from infancy to the third year, then steadily declines until the twenty-fifth year, when it rises slightly again. The mortality amongst cases under five years of age is twenty per cent., above that age it is seldom more than five per cent., so that the chance of recovery is least during infancy, and betters every year up to the twenty-fifth. In childhood, too, the death-rate from scarlatina is higher amongst males than females, but after the tenth year this order is reversed. Attacks of scarlet fever are of exceptional severity in certain predisposed families, and in children of tuberculous diathesis it frequently runs an unfavourable course. Unlike measles, however, this disease does not appear to attack the weakly and ill-fed with peculiar virulence, nor have overcrowding, or insanitary conditions generally, so prejudicial an effect. It is true that in overcrowded hospital wards the cases are of a graver nature and complications are more common, but in general there is no such marked distinction between the type of disease amongst rich and poor as is seen in measles. Epidemics vary much in type, but attacks are always more fatal in the winter months than in spring or autumn. It is possible that this may be accounted for by the increased risk of chill during the colder season. Important aid to successful prognosis is only to be found by careful attention to the symptoms and physical signs of each individual case.

The extent and intensity of the rash affords no absolute criterion of the seriousness of the attack. If, however, the rash be of a livid hue, purple, or hæmorrhagic, occurring in patches, with wide, clear, intervening areas, a grave prognosis should be given. This is more especially the case when there is much faucial implication. Indeed, to some extent, the degree of faucial trouble may be regarded as indicative of the gravity of the case. With severe angina is generally associated high temperature, which, if it persist beyond the tenth day, or rise still higher at the end of the first week, should be a cause for anxiety. High temperature alone is not necessarily dangerous, and recovery has taken place after the thermometer had registered 110 deg. Fahr.

Cases which terminate fatally are often marked in the early stage by delirium and coma, with persistent bilious vomiting and diarrhœa. Later, a feeble pulse and cold extremities are symptoms of cardiac failure. Such cases may terminate in forty-eight hours from the commencement of the attack.

A small, frequent, and compressible pulse, especially if its rate exceed one hundred and fifty beats per minute, is always an unfavourable symptom. Other signs indicative of failing power are restlessness, duskiness of the skin, profuse sweating, sordes, and dilatation of the pupil.

The occurrence of the various complications and sequelæ are more likely to prolong the patient's convalescence or to leave some permanent injury than to bring the case to a rapidly fatal termination.

The presence of a greater or smaller quantity of albumen in the urine may be, to a certain extent, regarded as a measure of the quantity of poison in the system. It is often associated with adenitis, and, as a rule terminates favourably. Nephritis is rarely immediately fatal, although it may leave the sufferer a more or less permanent invalid. Caiger, in his analysis of a thousand cases of scarlet fever, only found one death from nephritis.

Otorrhœa and rhinorrhœa may materially prolong the illness; unless, however, the mastoid cells be attacked, life is not endangered. These secondary discharges occur more frequently and are most persistent in patients with hypertrophied tonsils and adenoid vegetations.

The scarlatina that follows surgical operations is generally slight in nature, and if strict antiseptic precautions are observed, the wounds heal with accustomed readiness.

If other diseases be superadded to the scarlet fever, the prognosis is not so favourable. Measles, and diphtheria especially, may cause the condition of the patient to be one of great jeopardy. One attack of scarlatina does not necessarily confer immunity, nor is the second attack always of lessened severity. For instance, a brother and sister, both of whom had suffered previously, were, after an interval of sixteen years, again attacked. Both illnesses were of exceptional severity, and that of the sister terminated fatally. Relapses, however, are said to be of a milder character than the primary sickness, and, as a rule, they are so. Caiger, however, reports two cases of fatal relapse.

In the pregnant woman, scarlatina, especially if severe in type, may induce abortion. Under more favourable circumstances, however, gestation is uninterrupted. The dangers following natural labour, or abortion during this disease may be reduced to a minimum by the adoption of rigid antisepsis, for it seems probable that in all cases the septic material is introduced from without, either by the means of instruments or in the course of digital examinations.

Prognosis in scarlet fever must always be a matter of difficulty, for the disease is subject to marked variations in type, and in certain families there would seem to be a predisposition to a fatal issue. When, however, the disease is ushered in by convulsions, or by other marked nervous symptoms, such as delirium or coma, and when from the commencement there are signs of cardiac enfeeblement and depression, the worst result may be anticipated.

